# Under control: A WRKY transcription factor positively regulates the expression of a helper NLR immune receptor in *Nicotiana benthamiana*

**DOI:** 10.1093/plphys/kiaf445

**Published:** 2025-09-30

**Authors:** Josephine H R Maidment

**Affiliations:** Assistant Features Editor, Plant Physiology, American Society of Plant Biologists; PHIM Plant Health Institute, University of Montpellier, INRAE, CIRAD, Institut Agro, IRD, 34980 Montpellier, France; Centre de Biologie Structurale, INSERM U 1054, CNRS UMR 5048, Université de Montpellier, 34090 Montpellier, France

To defend themselves against pathogens, plants have an innate immune system that can be conceptually divided into 2 layers (reviewed by Bentham et al. 2020). Cell surface receptors recognize molecular signatures of pathogen attack in the extracellular space, while cytoplasmic nucleotide-binding leucine-rich repeat (NLR) receptor proteins detect molecules, known as *effectors*, which are secreted by the pathogen into the plant cell to promote infection. Together, these 2 classes of receptors coordinate defense responses to restrict the spread and impact of a pathogen. Basal immune responses, predominantly conditioned by cell surface receptors, include production of reactive oxygen species, callose deposition, synthesis of antimicrobial compounds, and upregulation of defense genes. By contrast, effector-triggered immunity frequently culminates in localized cell death, known as the *hypersensitive response*. This extreme outcome serves to restrict the spread of a pathogen to neighboring cells.

In some cases, a single NLR protein can detect an effector and activate immunity. However, certain NLRs have become specialized as effector *sensors* and require *helper* NLRs to trigger plant defenses (reviewed by Contreras et al. 2023). One such helper NLR is NRG1, which is required by a subset of sensor NLR proteins containing an N-terminal TIR domain (TNLs) to induce immune responses following effector recognition. Structural and biochemical studies have elucidated the molecular detail of how effector recognition by a sensor TNL leads to activation and oligomerization of NRG1 (Lapin et al. 2019; Jacob et al. 2021; Jia et al. 2022; Ibrahim et al. 2024). Interestingly, NRG1 oligomerization requires input from cell surface receptors (Feehan et al. 2023), highlighting that NRG1 is an important node integrating inputs from both layers of the plant immune system. However, while our mechanistic understanding of NRG1 activation has advanced in recent years, comparatively little is known about the transcriptional regulation of NRG1.

WRKY transcription factors (TFs) are abundant in plant genomes. They are so named for a signature WRKYGQK motif found in their DNA binding domain, which binds conserved W-box *cis* elements in promoters of target genes to modulate gene expression. WRKY TFs have regulatory roles in plant development as well as abiotic and biotic stress responses. Several have well-defined functions in regulating expression of defense-related genes and influencing disease outcomes (reviewed by Javed and Gao 2023).

In a recent study published in *Plant Physiology*, Wu et al. (2025) identified a WRKY TF, WRKY7, which regulates the expression of *NRG1* in *Nicotiana benthamiana*. Wu et al. performed DNA pulldown assays with the *NRG1* promoter to identify candidate transcriptional regulators. Transient overexpression of candidates in *N. benthamiana* subsequently identified 7 proteins that limited the spread of turnip mosaic virus, suggesting that these proteins are positive regulators of plant immunity. Upon observing that the *NRG1* promoter contained W-box motifs, the authors selected one of these proteins, WRKY7, for further investigation.

Using electrophoretic mobility shift and yeast 1-hybrid assays, the authors demonstrated direct binding of WRKY7 to the promoter of *NRG1*. In addition, they investigated the role of WRKY7 in modulating *NRG1* expression in vivo using a transient dual luciferase reporter assay, with firefly luciferase expression driven by the *NRG1* promoter. Overexpression of WRKY7 increased firefly luciferase activity, but mutation of the W-box motifs in the *NRG1* promoter abolished this increase, supporting the conclusion that WRKY7 binds W-box motifs in the *NRG1* promoter to enhance *NRG1* expression.

After observing that infection by either tobacco mosaic virus (TMV) or turnip mosaic virus induced *WRKY7* and *NRG1* expression but without activating cell death, Wu et al. investigated the effect of WRKY7 on basal immunity. Silencing *WRKY7* led to a reduction in *NRG1* transcription and to an increase in susceptibility to GFP-TMV, evidenced by increased viral coat protein levels as compared with wild type (WT) plants. This indicates that WRKY7 has a role in modulating basal immunity, potentially through regulation of *NRG1* expression.

To explore the contribution of WRKY7 to effector-triggered immunity mediated by the TNLs N (which recognizes the TMV replicase p50) and Roq1 (which detects the effector XopQ secreted by the bacterial pathogen *Xanthomonas euvesicatoria*), Wu et al. silenced *WRKY7* in *N. benthamiana* plants carrying *N* or *Roq1*. Following transient expression of p50 or XopQ, reduced cell death, indicating a reduced immune response, was observed in *WRKY7*-silenced plants as compared with WT plants. *WRKY7*-silenced plants carrying *N* or *Roq1* were more susceptible to TMV or *X. euvesicatoria*, respectively, than WT plants, further implicating WRKY7 in effector-triggered immunity.

Finally, Wu et al. observed that *WRKY7* expression is strongly induced during effector-triggered immunity. Interestingly, induction of *WRKY7* expression was compromised in *NRG1* knockout plants, suggesting that NRG1 and WRKY7 are engaged in a positive feedback loop, with each amplifying the expression of the other. Positive feedback loops can rapidly and robustly amplify responses to stimuli and, in the absence of additional regulatory mechanisms, irreversibly determine cell fate. Effector-triggered immunity typically culminates in programmed cell death to restrict the spread of the pathogen; the WRKY7-NRG1 circuit may contribute to a swift induction of cell death and resistance.

In this work, Wu et al. shed light on the role of WRKY7 in regulating *NRG1* expression and modulating basal and effector-triggered immunity (Fig.). The authors note that there appear to be multiple *cis* elements upstream of the *NRG1* coding region and that *NRG1* expression is likely modulated by numerous TFs to integrate different signals. Additionally, other WRKY TFs may be involved in regulation of *NRG1* through the identified W-box motifs in the *NRG1* promoter. Conversely, it remains to be explored whether WRKY7 regulates expression of other components of the signaling pathway leading to NRG1 activation or the plant immune system more broadly.

**Figure.**
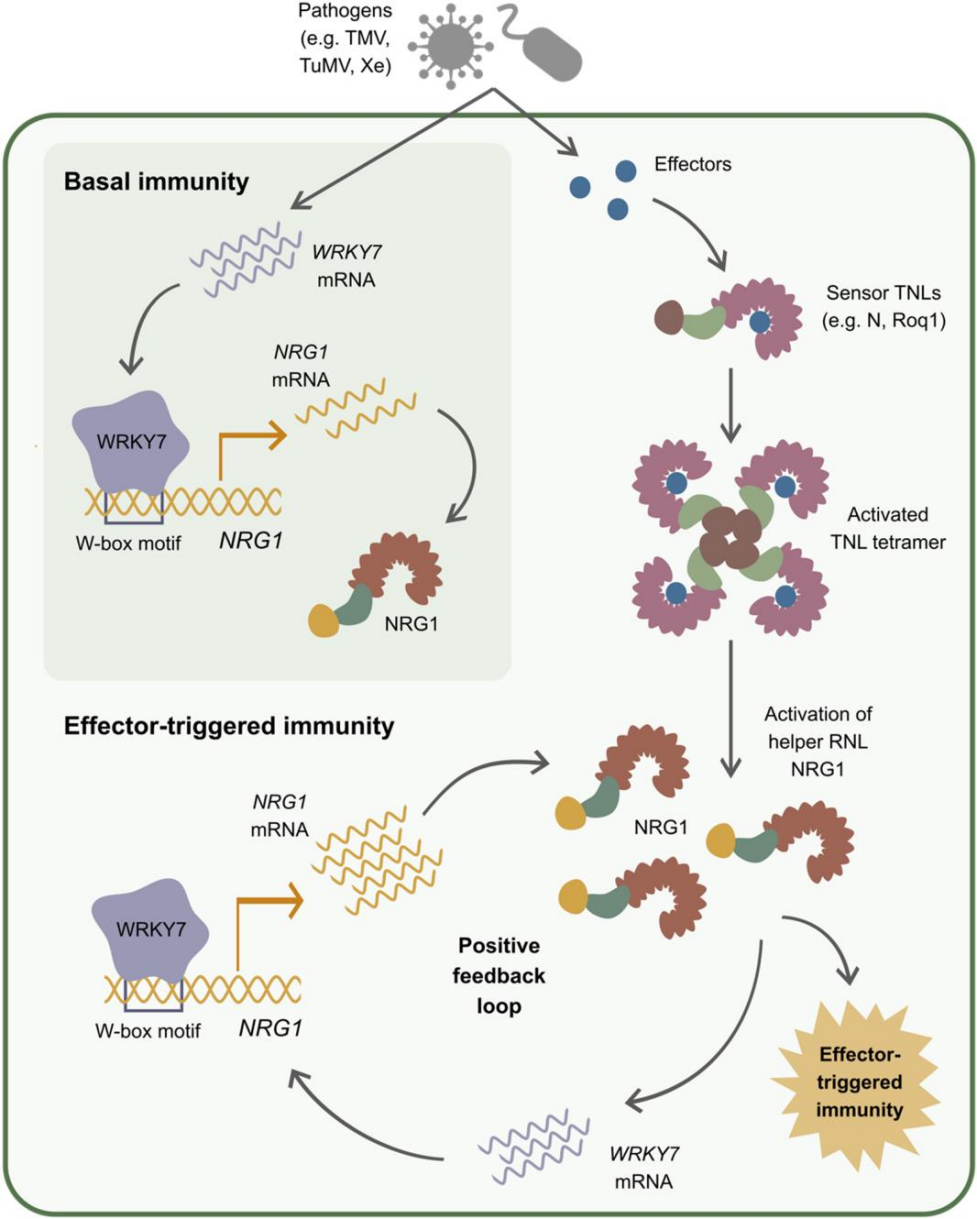
Model proposed by Wu et al. (2025) for the role of WRKY7 in plant innate immunity. During activation of basal plant immunity, expression of *WRKY7* and *NRG1* is induced. When TNL receptors detect cognate effectors, they assemble into a tetrameric holoenzyme, which ultimately leads to activation of NRG1, leading to effector-triggered immunity. Through a currently unknown mechanism, NRG1 enhances *WRKY7* expression, and in turn, WRKY7 positively regulates *NRG1* expression, resulting in a positive feedback loop. Adapted from Figure 6 of Wu et al. (2025).

While untangling the intricate network of regulation of NLR expression requires significant work, it is a highly useful endeavor. Transfer of NLR proteins between plant species has the potential to confer novel pathogen resistance in agriculturally important crops. Furthermore, a combination of structural data and developments in artificial intelligence for protein modeling and design has accelerated efforts to engineer synthetic NLR proteins with new-to-nature recognition profiles (reviewed by Zdrzałek et al. 2023). However, overexpression of some NLR proteins leads to deleterious phenotypes, such as spontaneous cell death and restricted growth, while insufficient expression prevents robust activation of defense. An improved understanding of the transcriptional, translational, and posttranslational regulation of NLR proteins, such as NRG1, may aid efforts to introduce NLR genes into crops. This study by Wu et al. represents a timely contribution to our understanding of the transcriptional regulation of an important component of the plant immune system.

## Data Availability

No new data were generated or analysed for this article.
